# Milestones in pathology—from histology to molecular biology

**DOI:** 10.1007/s12254-016-0307-z

**Published:** 2017-01-09

**Authors:** Leonhard Müllauer

**Affiliations:** Department of Pathology, Medical University Vienna, Währinger Gürtel 18–20, 1090 Vienna, Austria

**Keywords:** Molecular pathology, Next generation sequencing, Targeted therapy, Liquid biopsy, Biobank

## Abstract

Autopsy, histology and cytology have been and histology and cytology still are the main diagnostic tools in surgical pathology. During the last two decades molecular biology gradually has extended the diagnostic armamentarium. In tumor pathology molecular biology techniques are used to diagnose and subclassify tumors, predict response to therapies and identify therapeutic targets. Molecular pathology has evolved into a novel focus of clinical pathology and transforms the historically morphology based discipline. Traditional pathology and molecular pathology combine and guide tumor therapy.

Pathologists diagnose neoplastic diseases from tissue biopsies, surgical specimens and cytology aspirates. They employ as standard methods light microscopy of hematoxylin and eosin stained tissue slides and Giemsa or Papanicolaou stained cytologic smears. During the last half century the conventional light microscopy has been supplemented by immunohistology with expanding panels of antibodies [1]. The tasks for classic tumor pathology are tumor classification, grading and staging. There is a growing demand for molecular tumor subclassification and information on prognosis, response to therapy and molecular therapeutic targets. These demands are increasingly covered by molecular pathology and require an extended spectrum of methods. These encompass molecular biology techniques like polymerase chain reaction (PCR), DNA and RNA sequencing, flurorescence in situ hybridization and gene array assays.

The success of therapies that target genetic alterations has initially been demonstrated by imatinib treatment of chronic myeloid leukemia with BCR-ABL gene fusion [2]. In solid tumors the response of ERBB2 gene amplified breast carcinomas to the anti-ERBB2 antibody trastuzumab is a further landmark in the development of targeted therapies [3]. The efficacy of tyrosine kinase inhibitors erlotinib and gefitinib in the treatment of EGFR-mutated lung adenocarcinomas was a further boost for the concept of targeted therapy [4, 5] and forced pathology departments to implement EGFR mutation testing. In the meantime the number of therapeutic targets and the requirements for genetic testing have increased (Table 1).

**Table 1:** Genes routinely analysed for mutations in solid tumors in diagnostic molecular pathology (selection)

Tumor	Altered genes	Therapy (selection)
Lung adenocarcinoma	EGFR	Gefitinib, erlotinib, afatinib, osimertinib
	ALK	Crizotinib, ceritinib
	ROS1	Crizotinib
	MET exon 14	Crizotinib
Gastrointestinal stroma tumor (GIST)	KIT	Imatinib, sunitinib
	PDGFRA	Imatinib, sunitinib
Colorectal carcinoma	KRAS, NRAS, BRAF, MSI^a^	Cetuximab, panitumumab, immune checkpoint inhibitor
Malignant melanoma	BRAF	Vemurafenib, dabrafenib, trametinib, cobimetinib
	KIT	Imatinib, sunitinib, dasatinib
Breast carcinoma	HER2	Trastuzumab, pertuzumab
Ovarian carcinoma; triple negative breast carcinoma	BRCA1/2	Olaparib
Medullary thyroid cancer	RET	Vandetanib

^a^Microsatellite instability

The development of molecular tumor subclassifications and targeted therapies was facilitated by an improved knowledge of genetic aberrations. Although cancer research has achieved great successes during the last decades, e. g. the identification of oncogenes and tumor suppressor genes, the genetic characterisation of tumors has gained a tremendous speed during the last 10 years by the development of next generation sequencing (NGS) [6]. This technology brought the capacity to analyse the genomes of a large number of tumors of different entities [7–9]. NGS enables the simultaneous and rapid sequencing of millions of DNA molecules at reduced costs. The first sequencing of the human genome with the traditional Sanger technology required approximately 13 years at a cost of about 3 bn US$ [10, 11]. NGS can analyse a human genome within a week at a cost close to 1000 US$. The currently most potent Sanger-based sequencers analyse 1–2 megabases per day, whereas even small bench top NGS instruments sequence 3–15 gigabases per day. A further advantage of NGS is the versatility of applications. In addition to DNA sequence determination it is applicable for the detection of amplifications, deletions, gene fusions, DNA methylation and gene expression. Furthermore NGS is scalable; it can be adjusted to gene panels, the human exome (≈1.5% of the genome, ≈4.5 × 10^7^ bases) or the whole genome (≈3 × 10^9^ bases).

The massive genomic characterisation of tumors has been led by the The Cancer Genome Atlas (TCGA; http://cancergenome.nih.gov/) and the International Cancer Genome Consortium (ICGC; https://icgc.org/). TCGA has studied more than 10,000 specimens of 33 different tumor entities on the DNA, RNA and epigenome level [12]. The ICGC is a joint effort of 22 countries with the aim to analyse a total of 25,000 specimens from 50 different tumor entities. The results of TCGA and ICGC are freely available to the public (https://gdc.cancer.gov/ and https://dcc.icgc.org/).

It is often difficult to distinguish mutations from polymorphisms. Therefore efforts that identify polymorphisms are important for tumor diagnostics. The 1000 Genomes Project (http://www.1000genomes.org/) has generated a catalogue of genetic variants from 2504 people of 26 populations in 5 continental regions with the aim to identify most of the polymorphisms that occur at a frequency of at least 1% [13]. The International Genome Sample Resource (http://www.internationalgenome.org/home) continues the 1000 Genomes Project. A further valuable initiative for the interpretation of tumor genome sequences are the databases of Short Genetic Variations (dbSNP) and Genomic Structural Variations (dbVar) of the NCBI (https://www.ncbi.nlm.nih.gov/snp; https://www.ncbi.nlm.nih.gov/dbvar).

At present single gene analysis with mutation-specific PCR and Sanger- or pyrosequencing [14] predominates in diagnostic molecular pathology. However gene panel sequencing with NGS increases rapidly. These gene panels are either adapted for a tumor entity and encompass the most frequent predictive and prognostic mutations for that entity or represent larger pan-cancer panels that cover most of the known tumor driver genes [15].

The analysis of NGS data is challenging. In recent years commercially available software tools have improved and permit individuals with basic bioinformatic proficiency to interpret NGS data. The more demanding NGS applications like transcriptome sequencing or gene copy analysis however require bioinformatic professionals. Therefore pathology departments have to recruit and integrate bioinformaticians to master the increasing demand for genetic tumor profiling.

Currently molecular pathology adopts also the analysis of cell-free DNA (cfDNA), which is released by dying normal or tumor cells into the blood [16]. The analysis of cfDNA can substitute for a tissue biopsy in certain indications. Therefore the expression “liquid biopsy” has been coined. However, it has to be emphasized that at least for the initial tumor diagnosis a tissue biopsy is essential. The biggest challenge in the analysis of cell-free tumor DNA (ctDNA) is the often low frequency of mutated alleles in cfDNA. The amount of ctDNA is variable and ranges from 0.01% to more than 50% of the whole cfDNA. An advantage of liquid biopsy is the low burden for the patient as compared to tissue biopsy with a blood draw of 5 to 10 ml. Furthermore, tumors possess an intratumoral and intermetastatic genetic heterogeneity [17]. A tissue biopsy therefore often does not capture the whole spectrum of genetic changes of a tumor. The ctDNA however may better represent the genetic composition of different tumor compartments. A further advantage is that DNA modifications caused by formalin fixation of tissue and the resulting artefacts in DNA sequencing [18] are not present in ctDNA. A liquid biopsy can be utilised for various indications in tumor patients (Table 2), although most applications are still in clinical validation. The major methods to detect mutations in ctDNA are allele-specific PCR and NGS. PCR methods with a particularly high sensitivity are necessary, such as droplet digital PCR, for which a lower detection limit of 0.01% mutated allele frequency has been reported [19]. NGS protocols have to be adapted for liquid biopsy, e. g. by tagging DNA fragments with unique molecular barcodes [20], to increase the sensitivity of mutation detection. In tumor pathology at present only the detection of resistance mutations, in particular in adenocarcinomas of the lung, is clinical routine. EGFR-mutated lung adenocarcinomas with secondary resistance to tyrosine kinase inhibitors exhibit in approximately 60% a secondary T790M EGFR mutation. This mutation can be detected in 70% of cases in ctDNA [21]. Patients with this mutation can profit from third generation EGFR inhibitors, such as osimertinib, that are able to block T790M mutated EGFR.

**Table 2:** Applications of liquid biopsy in tumor diagnostics

Indications
Identification of resistance mutations
Identification of targets for therapy
Monitoring of tumor load
Monitoring early response to therapy
Monitoring of “minimal residual disease”
Assessment of molecular tumor heterogeneity
Early tumor detection

Circulating tumor cells (CTC) are very scarce in the blood. Mostly less than 10 CTC are present in 1 ml blood in patients with metastasis [22]. In comparison to ctDNA the utilisation of CTC for the detection of somatic mutations is not clinical routine diagnostics because of the very low amount of available DNA and the high equipment and technical skill requirements.

The challenges in molecular pathology require a new type of pathologists. The pathologists of the future need to combine morphological methods with practical and theoretical knowledge in genetics, cell biology, biochemistry and bioinformatics. The university departments of pathology will have to fulfill a leading role in training surgical and molecular pathology. The establishment of molecular pathology requires space, equipment and personal. Their financing is particularly difficult for smaller institutes. Therefore not all institutes will be able to offer a broad spectrum of molecular pathology diagnostics. A concentration in larger institutes, mainly university departments, that serve as reference centers, is likely.

Molecular pathology tests are mostly performed with formalin fixed and paraffin embedded tissues (FFPE). The advantage is that the tissue blocks generated for histology can also be used for molecular tests. A disadvantage is the fragmentation and chemical modification of DNA by formalin [18]. Exome, whole genome and transcriptome sequencing have become feasible with FFPE tissue; however for these applications fresh, unfixed tissue is still advantageous. Therefore departments of pathology should establish biobanks for the preservation of frozen, unfixed tissues.

The future will bring an increase in molecular pathology testing. The pace will be determined by the availability of effective targeted drugs. Gene panels will replace single gene analysis. Exome, transcriptome and perhaps epigenome analysis will be widely used and the applications of liquid biopsy will expand. Furthermore proteome analysis and in vitro drug susceptibility testing may become further tasks. Molecular pathology will transform the classic morphology-based pathology. Pathologists will become pilots for precision cancer therapy through their unique ability to combine morphological and molecular findings.
